# Development of Eco-Efficient Recycled Concrete Incorporating Steel Slag, Ground-Granulated Blast-Furnace Slag, and Fiber: Mechanical Properties and Strength Prediction Based on Artificial Intelligence Techniques

**DOI:** 10.3390/ma19132752

**Published:** 2026-06-28

**Authors:** Shaofeng Zhang, Xue Wang, Ditao Niu, Yan Wang, Daming Luo

**Affiliations:** 1 Shaanxi Railway Institute, Weinan 714000, China; 2 College of Civil Engineering, Xi’an University of Architecture and Technology, Xi’an 710055, China; 3 College of Materials Science and Engineering, Xi’an University of Architecture and Technology, Xi’an 710055, China

**Keywords:** recycled aggregate concrete, steel slag, ground granulated blast-furnace slag, PP fiber, artificial neural network

## Abstract

Reusing industrial byproducts to prepare recycled aggregate concrete (RAC) is a sustainable approach that can protect the ecological environment. This study tested the possibility of preparing an eco-efficient recycled concrete containing steel slag (SS), ground-granulated blast-furnace slag (GGBS), and polypropylene (PP) fibers to avoid resource waste and depletion and decrease CO_2_ emissions. To this end, 12 mix proportions were designed to analyze the effects of SS, GGBS, and PP fibers on the macro- and micro-performances of the developed RAC. The experimental results showed that increasing the SS content decreased the RAC mechanical strength, whereas partially substituting SS with GGBS in the RAC improved the mechanical properties, especially at a later stage. Adding PP fibers to the RAC containing SS and GGBS significantly increased the splitting tensile strength. However, it had little effect on the compressive strength as the PP fiber content was less than 0.6%. The microscopic experiment revealed that adding GGBS promoted the degree of hydration of SS, reduced the Ca (OH)_2_ content, made the ITZ structure more compact, and optimized the pore characteristics of the RAC. Furthermore, according to the raw materials and results of mechanical properties, a hybrid Genetic Algorithm/Artificial Neural Network (GA-ANN) technique was proposed to predict the compressive strength of the RAC containing SS, GGBS, and PP fibers. We found that the proposed GA-ANN model effectively predicts the compressive strength. The findings of this study demonstrate that preparing RAC incorporating SS, GGBS, and PP fibers is promising for the reuse of industrial byproducts and construction waste.

## 1. Introduction

The World Business Council for Sustainable Development defines the concept of eco-efficiency [1] as “*the preparation of products satisfy the requirement of humankind with living quality, while gradually decreasing their environmental implication and consumption of raw materials throughout their life cycle, to a level compatible with the capacity of the planet*”. According to the Council, eco-efficient cement-based materials in the construction industry exhibit a low environmental impact and high performance. As one of the main eco-efficient cement-based materials, RAC is a novel low-carbon construction composite material that can save 60–70% of natural materials and decrease 15–20% of CO_2_ emissions [2]. The use of RAC as a renewable resource in concrete could reduce the application of nonrenewable natural resources and decrease construction and demolition waste, contributing to both resource utilization and environmental protection. The mechanical, microstructural, and durability properties [3], structural behavior [4], and fire resistance [5] of RAC have been extensively studied worldwide since 2010. Although previous research indicated that the presence of adhered mortar in recycled aggregate (RA) increased the number of ITZs on RAC, leading to lower mechanical and durability properties in comparison to conventional concrete [6], some enhancement measures [7], including the Two-Stage Mixing Approach pretreatment and the use of pozzolanic material, have been applied to improve the performance of RAC. The results also demonstrate that applying RAC in practical projects can meet the design and specification requirements, leading to its wide application in many countries in recent years.

Replacing cement with supplementary cementitious materials (SCMs) during the preparation of RAC reduces carbon emissions and improves the green footprint in eco-efficient concrete production. The most widely used SCMs [8] include ground-granulated blast-furnace slag (GGBS), marble powder, fly ash, silica fume, iron tailing powder, and metakaolin, which can improve the chemical/physical characteristics of RAC owing to their pozzolanic reaction. However, the quantity of SCMs is limited, and their prices are relatively high, due to geographical and technological limitations [9]. To prepare sustainable RAC, applying other industrial byproducts, such as steel slag (SS), is a promising approach as it can reduce CO_2_ emissions in the cement production process and realize the application of waste resources. SS is an industrial solid residue product of the steelmaking process and is believed to be an alternative mineral admixture obtained through mechanical grinding [10]. Owing to its wide availability [11], economy [12], hydraulic activity [10], and filling effect [13], the cementitious and macroscopic properties of SS—such as hydration products, mechanical properties, microstructure, soundness, and durability—have been widely investigated. The results of these investigations indicate that SS is a potential SCM for low-carbon cement-based materials. However, hydraulic activity was found to be significantly lower in the cooling mode. Previous studies [14,15,16] have shown that SS activity can be enhanced by with mechanical grinding, high-temperature, and chemical activation; however, the improvement effect is limited. Therefore, adding other SCMs to make up for the low activity of SS is an effective method for improving the SS utilization rate. Among the most commonly used SCMs, GGBS exhibits high activity and reacts with calcium hydroxide (Ca(OH)_2_) to generate calcium silicate hydrate (C-S-H gel), which facilitates SS dissolution and enhances RAC’s mechanical performance at an early stage. The enhancement of GGBS with aluminum silicate and an active calcium source [17] was shown to effectively complement the shorting of SS, which led to the production of C-S-H or C-A-S-H gel in RAC. Therefore, combining SS and GGBS in the preparation of RAC is a novel approach for developing eco-efficient RAC.

Meanwhile, fibers significantly enhance the post-cracking toughness and improve the energy absorption of the concrete materials. Su et al. [18] reported that adding polypropylene (PP) fibers not only inhibited the occurrence and expansion of cracks under various loads but also solved the problem of stress focus by reducing sharp corners or locally supplying strength concentration at the cracks. Two studies [19,20] reported that the mechanical behavior and durability of concrete could be markedly enhanced by the addition of PP fibers, due to the role of PP fibers in controlling the propagation of local cracks and relieving stress focus. The cost-effectiveness and environmentally friendly inorganic nature of PP fibers further promote its reinforcement application in concrete. Additionally, Faraj et al. [21] and Hossain et al. [22] studied the impact of PP fibers on the toughness and ductility of RAC and reported that PP fibers not only significantly improved the toughness and crack resistance but also provided good alkali resistance.

Moreover, it is time-consuming and expensive to investigate the mechanical characteristics of RAC in laboratories because of its complex and variable mixtures. Therefore, empirical relationship methods such as multiple linear and nonlinear regression are typically employed to predict RAC characteristics; however, they cannot reflect the relationships among raw materials, resulting in insufficient accuracy [23]. Hence, the application of artificial intelligence (AI) techniques has been proposed to precisely design and predict the performances of RAC containing different variables by training the obtained experimental results [24,25]. Artificial Neural Networks (ANNs), which simulate the learning function of the human brain by constructing the mapping relationships between multi-input and multi-output variables via in-depth training on sample datasets, have been widely used in the past decades to evaluate the mechanical properties of concrete [26,27]. An ANN is an effective alternative to regression analysis for data fitting and determining the underlying relationships between output and input data. It has advantages in extracting characteristic parameters and improving prediction accuracy without making assumptions about mathematical models [28,29]. However, ANNs are limited because they have inherent defects in initial weight sensitivity and require many calculations, leading to overfitting or local minimum values. To offset the shortcomings of ANNs, an innovative strategy was developed by combining a bionics-based method called Genetic Algorithm (GA) with an ANN model [30], which improves the applicability, stability, and accuracy of the ANN model by perfecting its algorithm structure. Some researchers employed a Genetic Algorithm-based Artificial Neural Network (GA-ANN) to evaluate the characteristics of mortar [31], concrete [32], and ultra-high-performance concrete [30] and analyzed the efficiency and sensitivity of GA-ANN and ANN. These previous investigations indicated that the efficiency and sensitivity of an GA-ANN model are superior to those of an ANN model.

At present, there is more research on the effects of SS, GGBS, and PP fibers on the mechanical properties of RAC, while research on the enhancement of the mechanical properties of RAC by applying hybrid SS, GGBS, and PP fibers is relatively limited. Few studies have attempted to predict the mechanical properties of RAC using the GA-ANN method. Based on the aforementioned investigations, this study aimed to develop an eco-efficient concrete, namely an RAC containing SS, GGBS, and PP fibers, to facilitate the application of industrial byproducts and construction waste. A systematic study was conducted to investigate the action mechanism of SS, GGBS, and PP fibers on the macro- and micro-performances of the developed RAC. In this case, 12 RAC mixtures containing 0–30% SS, 5–20% GGBS, and 0.2–0.8% PP fibers were developed. The mixtures were slumped before casting the specimens, and the compressive and splitting strengths were evaluated for up to 90 days. To illustrate the influencing mechanism, the evolutions of the hydration product, pore structure, and microstructure were investigated. The preliminary preparation of RAC generally involves numerous adaptation experiments, entailing substantial consumption of time, materials, and labor. The GA-ANN model was proposed to evaluate the compressive strength of the RAC containing SS, GGBS, and PP fibers.

## 2. Materials and Methods

### 2.1. Materials

**Figure 1:**
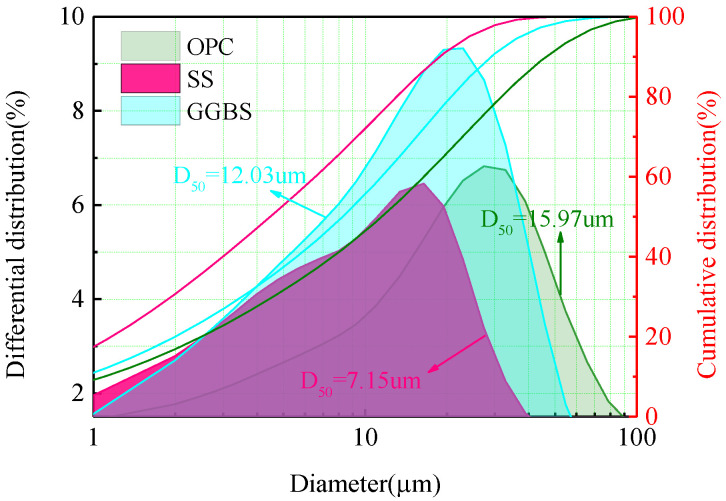
The particle size distribution of raw materials.

**Figure 2:**
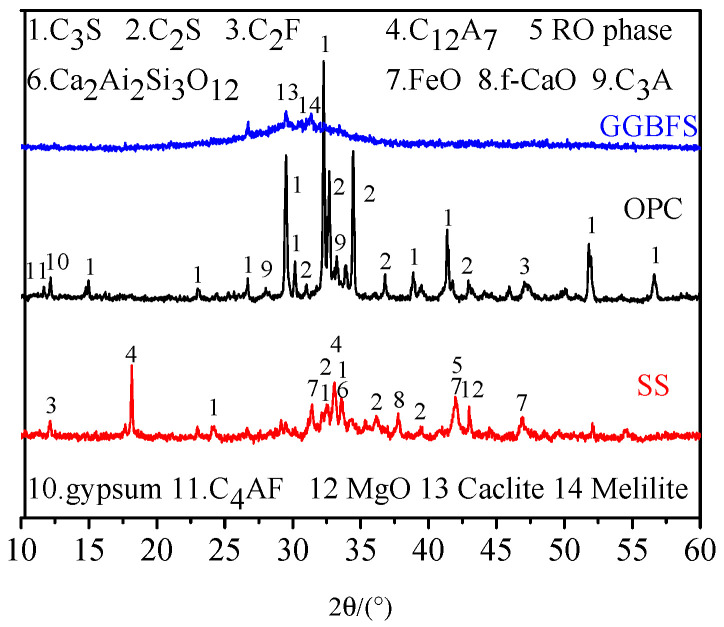
XRD patterns of raw materials.

P-O 42.5 Portland cement, SS, and GGBS were used for preparing the RAC. Their chemical compositions obtained with XRF and physical properties are presented in Table 1. Figure 1 plots the particle size distributions of OPC, SS, and GGBS measured using laser diffraction, and Figure 2 shows their mineral phase compositions identified with XRD analysis. The SS contained 2.32% f-MgO and 3.1% f-CaO, which met the requirements for volume stability according to a previous study [33]. River sand was used as fine aggregate (FA), and its maximum size, fineness modulus, and apparent density were 4.75 mm, 2.75, and 2.61 kg/m^3^, respectively. RA was purchased from Shaanxi Longfeng Ecological Stone Industry Co., Ltd. (Xi’an, China), with a crushing index of 14.6, a diameter of 4.75–20 mm, and an apparent density of 2.468 kg/m^3^. The length, diameter, density, elastic modulus, and tensile strength of the PP fibers were 19 mm, 30 um, 0.91 g/cm^3^, 0.3 GPa, and 270 MPa, respectively. The ordinary tap water and polycarboxylic acid superplasticizer (PBS) was used for mixing. Based on previous study [33], the water glass (WG) with 8.36% Na_2_O, 27.22% SiO_2_, and 64.42% H_2_O was used to stimulate the activity of SS. The modulus of WG was adjusted to 1.2 by adding NaOH. The dosage of the WG was chosen to be 4% of SS and GGBS (by weight).

### 2.2. Specimen Preparation

**Figure 3:**
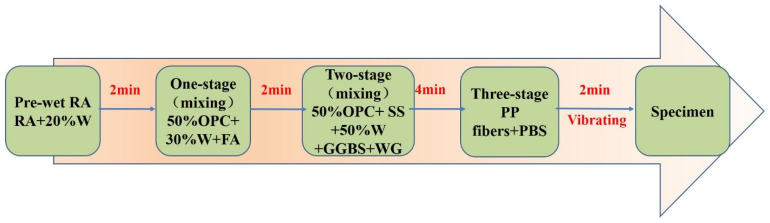
Schematic diagram of mixing procedure for RAC.

According to the standard “Steel slag powder for use in cement and concrete (GB-T 20491-2017)” [34], the proportion of SS can range from 0% to 30%. Based on studies of examining GGBS and PP fibers [17,19], the proportion of GGBS can range from 0% to 20% and the volume content of PP fibers is 0.2~0.8%. A total of 12 mixtures with a water binder of 0.42 was designed to analyze the influence of SS, GGBS, and PP fibers on the behavior of the developed RAC. Table 2 presents the details of the RAC. The fresh RAC specimens were cast into molds with 100 mm × 100 mm × 100 mm size for strength testing. The WG and NaOH were dissolved in water before test preparation. The RAC specimens were prepared by pre-wetting and secondary mixing; the mixing process is schematically illustrated in Figure 3. The slump test was conducted on each mixture using a slump cone based on ASTM C230 [35] prior to preparing the specimens. All specimens were maintained in a standard curing chamber until the day of testing.

### 2.3. Testing Method

#### 2.3.1. Mechanical Performance

The compressive and splitting tensile strengths on the designed days were tested according to ASTM C39 [36] and C496 standards [37], respectively. The average values obtained from three cubic specimens were used to evaluate the impact of SS/GGBS and PP fibers on the mechanical properties of the RAC.

#### 2.3.2. Microscopic Analysis

At designated ages, the pastes were soaked in a centrifugation tube with cold isopropanol to suspend the hydration. The pastes were dried in a desiccator at 50 ± 2 °C over 24 h and then used to prepare the samples for microscopic analysis. The mercury intrusion porosimeter (MIP) test (Auto Pore IV-9500, pore size range: 3 nm–360 µm, Micromeritics Instrument Corporation, Norcross, GA, USA) was used to investigate the pore size distribution and structure of the RAC. Microstructural characteristics were observed using a GEM-500 instrument (Werfen, Barcelona, Spain). The hydration product of the paste incorporating SS and GGBS was studied using XRD and TG analyses. XRD was measured on a Bruker D8 8 Discovery instrument (Bruker, Billerica, MA, USA) with a 5–60° scanning angle in steps of 0.02°. TG was conducted with an STA 449F5 thermogravimetry instrument (NETZSCH-Gerätebau GmbH, Selb, Germany) under a N_2_ atmosphere from 30 to 900 °C at a heating rate of 10 °C/min.

### 2.4. Key Steps of Building a GA-ANN Model

**Figure 4:**
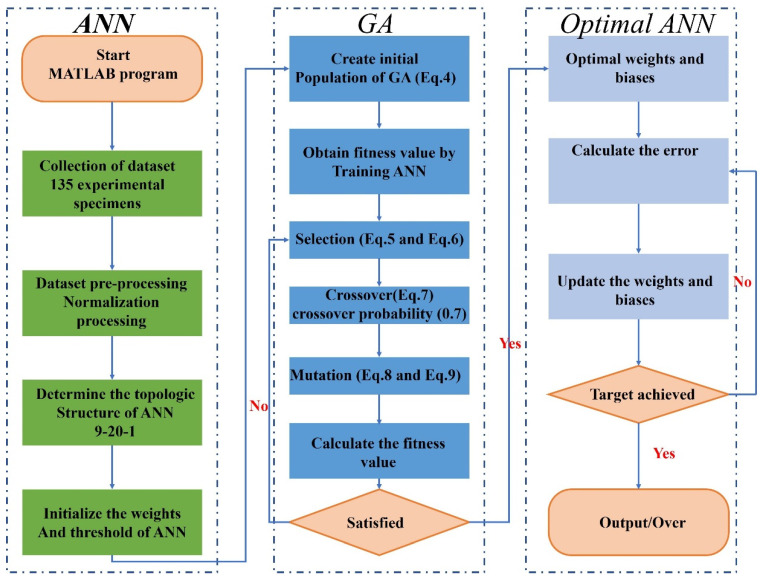
Calculation ideas of GA-ANN neural network.

The GA-ANN model for compressive strength prediction, which was mainly inspired by genetics and bionics, was created and trained using the MATLAB2024 program [31,32]. Generally, a Genetic Algorithm (GA) is a probabilistic adaptive iterative algorithm, with a global search ability, that is not limited by spatial information. However, the initial weight and threshold of an ANN are randomly generated, which quickly causes a local extremum and overfitting. Therefore, the GA was applied to complement the algorithm structure of the ANN by finding the optimal or approximate optimal solution in multiple regions of the global solution space, as presented in Figure 4. The algorithm implementation and fundamental principle of the GA-ANN model can be illustrated using the following steps:

(1) Original dataset collection and preprocess: Select the inputs, including OPC (*x*_1_), SS (*x*_2_), GGBS (*x*_3_), FA (*x*_4_), RA (*x*_5_), water-to-cement ratio W/C (*x*_6_), PP (*x*_7_), and sand ratio (the percentage of fine aggregate in fine aggregate and coarse aggregate, *x*_8_), and outputs (compressive strength, *Y_i_*) to establish a function *Y_i_* = *f* (*x*_1_, *x*_2_, *x*_3_, *x*_4_, *x*_5_, *x*_6_, *x*_7_, *x*_8_).

(1) $$Y=f\left(Z\right)=\frac{1}{1+e^{-Z}}$$

where *f* denotes the summation and activation functions, and *Z* (Equation (2)) denotes the weighted sum of the inputs adjusted according to bias factor (b).

**Figure 5:**
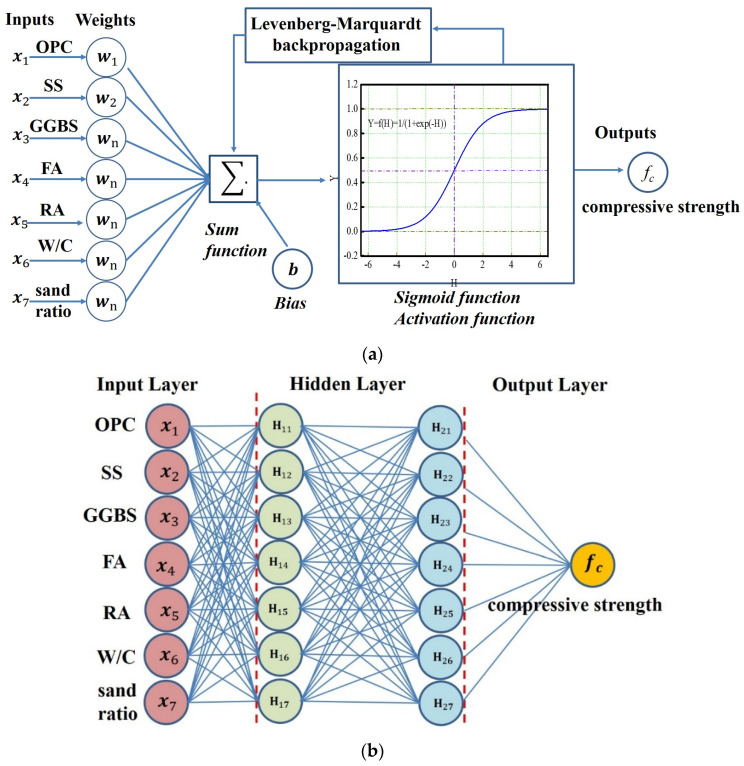
The algorithm structure diagram of ANN utilized in this study. (**a**) An artificial neuron in the MLP; (**b**) The multi-layer neural network model layout for *f_c_*.

(2) Determine the structure and algorithm of the ANN: First, an artificial neuron is obtained using Equation (1), which is applied to receive the weighted inputs and bias and to perform summation and activation functions to produce the outputs, as shown in Figure 5a.

(2) $$Z=\sum_{i=1}^{n}X_{i}W_{i}+b$$

where *X_i_* and *W_i_* are the input layer and corresponding weight, respectively.

Then, MLP (commonly referred to as multilayer perceptron) is utilized, as presented in Figure 5b. Finally, error backpropagation is used to adjust the weights, and the errors between the predicted outputs and actual values are established using Equation (3).

(3) △W=η×δ×ο

where *ο*, *δ*, *η*, and △*W* denote the output of the input neurons, the local gradient of the layer, the training rate, and the adjusted weight, respectively.

(3) Optimize ANN with GA.
  (a) Determine the fitness function, which includes the actual and predicted values as displayed in Equation (4):
    (4) $$F=k\left[\sum_{i=1}^{n}abs\left(o_{i}-y_{i}\right)\right]$$
    where *y_i_ o_i,_ n*, and *k* are the target results, simulated output value, number of output layer nodes, and coefficient, respectively.
  (b) Individual selection strategy: RWS (named here as roulette wheel selection) is used to determine the individual based on the fitting coefficient, and the selection procedure can be summarized as follows:
    (5) $$f_{i}=k/F_{i}$$
    (6) $$p_{i}=f_{i}/\sum_{j=1}^{N}f_{i}$$
    where *N*, *F_i_*, and *k* are the population number, individual fitness value, and coefficient, respectively.
  (c) Crossover and mutation: First, arithmetic crossover operators are adopted, and the crossover operation is as follows:
    (7) $$\{\begin{array}{c} a_{kj}=a_{kj}\left(1-b\right)+a_{ij}^{b}\\ a_{lj}=a_{lj}\left(1-b\right)+a_{kj}^{b} \end{array}$$
    where *a_kj_* is the *j* chromosome of the *k* individual and *a_lj_* is the *j* chromosome of the first individual.

Afterward, the mutation operation was performed in accordance with a certain probability variation, as illustrated in Equations (8) and (9):

(8) $$a_{ij}=\{\begin{array}{c} a_{ij}+\left(a_{ij}-a_{max}\right)\times f\left(g\right),\;\;r>0.5\\ a_{ij}+\left(a_{min}-a_{ij}\right)\times f(g),\;\;r\leq 0.5 \end{array}$$

(9) $$f\left(g\right)=r{\left(1-g/G_{max}\right)}^{2}$$

where *r* denotes a random digit ranging from 0 to 1, and *g* and *G_max_* denote the current number of iterations and maximum number of evolutionary generations, respectively.

(4) The compressive strength prediction model GA-ANN is developed by combining the ANN and GA.

## 3. Results and Discussion

### 3.1. Influence of SS/GGBS and PP Fibers on the Slump of RAC

**Figure 6:**
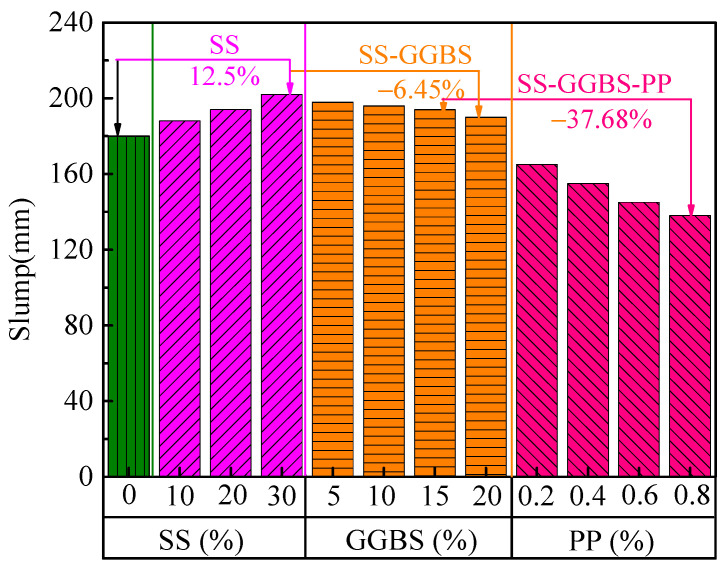
Slump of RAC with SS, GGBS and PP fibers.

The effects of SS, GGBS, and PP fibers on the slump of the RAC are shown in Figure 6. As displayed in Figure 6, the slump increased with an increase in SS content. Compared to the conventional RAC samples, when the cement was replaced with 10%, 20%, and 30% SS, the slump of the RAC samples increased by 4.45%, 7.78%, and 12.22%, respectively. Similar trends were reported in Ref. [38], which illustrated the relationship between cement substitution with SS and normal concrete workability. The observed experimental results are attributed to the SS having a low hydraulic activity at an early stage and fine spherical particles, thus acting as a lubricant and having filling effects. Replacing SS with 5–20% GGBS slightly decreased the slump of the RACSG, whose value varied from 202 mm to 190 mm. This may be because the surface area of the GGBS is significantly higher than that of the SS, thereby needing more water to attain the same fluidity [39]. Adding PP fibers at volume fractions of 0.2%, 0.4%, 0.6%, and 0.8% reduced the slump to 165, 155, 145, and 138 mm, respectively. The results indicated that adding PP fibers to RACSG remarkably decreased the workability of RACSGP. This is because the PP fibers absorb some free water and build a network skeleton in RACSGP, which not only renders the water film covering the RA thinner but also restrains the segregation of RACSGP [40,41].

### 3.2. Influence of SS/GGBS and PP Fibers on the Compressive Strength of RAC

**Figure 7:**
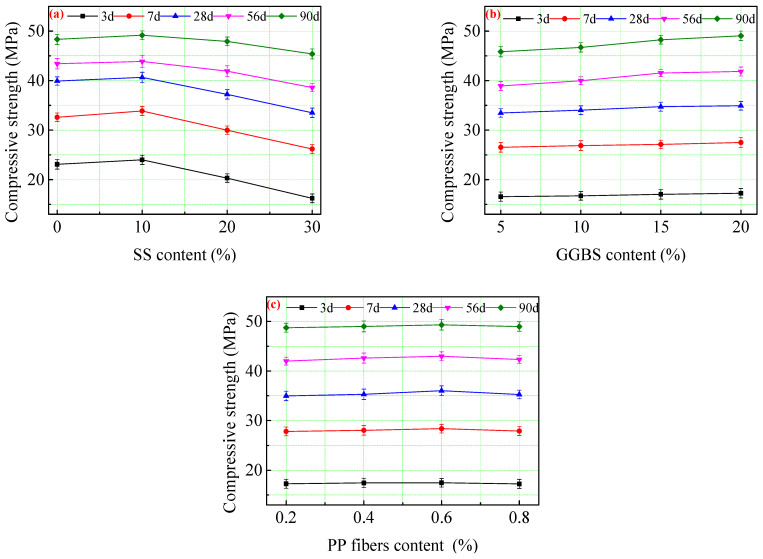
Compressive strength (**a**) RAC with SS (**b**) RAC with SS and GGBS (**c**) RAC with SS GGBS and PP fibers.

The compressive strength of the RAC with various mix proportions at different ages is shown in Figure 7. As illustrated in Figure 7a, substituting cement with SS led to a significant strength loss compared with the RAC specimen at 3~28 days, decreasing the variability by 12.21~28.61% at 3 days and 6.76~16.56% at 28 days, which was principally attributed to the limited reaction degree of SS [10]. The strength increase in RACS_10_ is traceable to the nucleation and filler effects of SS, which accelerate the dissolution and hydration reaction of the cement, resulting in the enhancement of the ITZ of RAC. However, when the substitution of cement with SS was beyond 10%, the negative influence on the strength of the inactive and inert components of SS surpassed the filler and nucleation effects of SS. As shown in Figure 7b, the partial substitution of SS with GGBS offers a comprehensive benefit in compressive strength but still lowers the strength at an early stage. Compared with RACS_30_, the compressive strength of RACS_25_G_5_, RACS_20_G_10_, RACS_15_G_15_, and RACS_10_G_20_ at 3 days increased by 2.09, 3.06%, 4.95%, and 6.32%, respectively. The compressive strength of RACS_25_G_5_, RACS_20_G_10_, RACS_15_G_15_, and RACS_10_G_20_ at 90 days increased by 7.21%, 9.09%, 10.25%, and 13.03%, respectively. These phenomena are due to the delayed pozzolanic effect of GGBS until a few weeks later [17], which promotes the hydration reaction of SS via the expending of Ca (OH)_2_ (CH) and the generation of a C-S-H gel, resulting in matrix densification as well as an enhancement in compressive strength.

As shown in Figure 7c, the compressive strength of RACSGP first increased and then decreased with an increase in PP fiber content. The introduction of PP fibers below 0.6% has little enhancement effect on RACSGP strength. Compared with RACS_15_G_15_, RACS_15_G_15_P_0.6_ exhibited higher compressive strength, which increased by 1.58%, 2.27%, 3.23% 3.82%, and 4.12% at 3, 7, 14, 28, 56, and 90 d, respectively. The improvement effect of 0.6% PP fibers at 90 d was the most significant, which was attributed to the pozzolanic effect of GGBS and fiber reinforcement theory. These findings are consistent with the conclusions drawn by Xue et al. [42] and Xu et al. [41] that the optimum PP fiber content with regard to the compressive strength was 0.6% (volume fraction). This experimental result is primarily attributed to the following: (i) The optimum PP fiber content in RACSGP can form a three-dimensional optionally dispersed PP fiber skeleton, which restricts the lateral deformation of the RAC under compression stress. (ii) When the PP fibers are broken or pulled out, the frictional force among the RACSGP interface and the PP fibers expend a portion of energy, which spontaneously postpones the destruction of the RACSGP. (iii) When the RACSGP is subjected to the load, the crack propagation inhibition of PP fibers is favorable for enhancing its strength [43], which will be discussed in Section 3.7.2.

### 3.3. Influence of SS/GGBS and PP Fibers on the Splitting Tensile Strength of RAC

**Figure 8:**
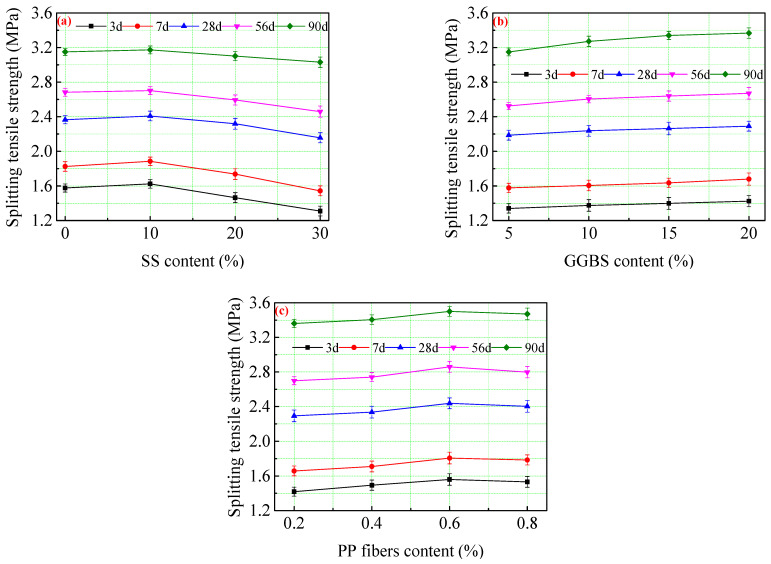
Splitting tensile strength (**a**) RAC with SS (**b**) RAC with SS and GGBS (**c**) RAC with SS GGBS and PP fibers.

The splitting tensile strength responses of the SS/GGBS- and PP fiber-incorporated RAC at different ages are presented in Figure 8. It is evident from Figure 8a that there is an analogous change tendency in both the splitting tensile and compressive strength of the RAC samples containing SS. The addition of SS decreased the early splitting tensile strength of RACS, except for the samples with 10% SS content. However, the splitting tensile strength of RAC containing SS developed more significantly at 90 days due to the hydration reaction of SS in a high alkaline matrix. For the same cement substitution level, the partial substitution of SS with GGBS was conducive to improving the strength. It is noteworthy that the splitting tensile strength of RACS_25_G_5_, RACS_20_G_10_, RACS_15_G_15_, and RACS_10_G_20_ at 90 days was 4.23%~11.13% higher than that of RACS_30_, due mainly to the chemical reaction of the GGBS which resulted in the generation of more C-S-H gels by consuming Ca (OH)_2_, thereby enhancing the matrix and ITZs of RACSG.

Figure 8c shows that incorporating PP fibers strengthened the splitting tensile strength of RACSGP. The incorporation of 0.2~0.6% PP fibers into RACSG led to an increase of 1.25~8.66% in the splitting tensile strength at 28 days. Compared with RACS_15_G_15_, the splitting tensile strength of RACS_15_G_15_P_0.6_ increased by 3.45% and 5.36%, respectively, at 56 and 90 days. However, the splitting tensile strength decreased by 0.85~2.1% at 3–90 days, including in samples containing 0.8% PP fibers, in comparison to RACS_15_G_15_P_0.6_. The introduction of PP fibers contributes to the ultimate tensile strain and minimizes the brittleness of RACSGP caused by the addition of SS and GGBS, as discussed in Section 3.7.2. The anti-cracking ability of the PP fibers also inhibits the generation and propagation of cracks to a certain extent when placing RACSGP under pressure. However, when an excessive amount of PP fibers is added, they may become clumped and unevenly dispersed, and more hydration products are needed. This negatively influences the cementation between the hydration product and RA and decreases the splitting tensile strength. To clarify the optimum PP fiber content for RACSGP, the experimental results were compared with the results obtained from previous studies. The results reported by Das et al. [44] and Zhou et al. [45] revealed that the optimum content of PP fibers was 0.6%, which effectively strengthened the tensile properties of RAC. Xu et al. [41] summarized the increment rate of the splitting tensile strength affected by PP fibers, which varied from 0 to 20.07% in previous experimental studies. By comparing the results of previous studies and the present study, it can be concluded that the optimum PP fiber content for RACSGP is 0.6%.

### 3.4. Modification of Hydration Products with SS/GGBS

**Figure 9:**
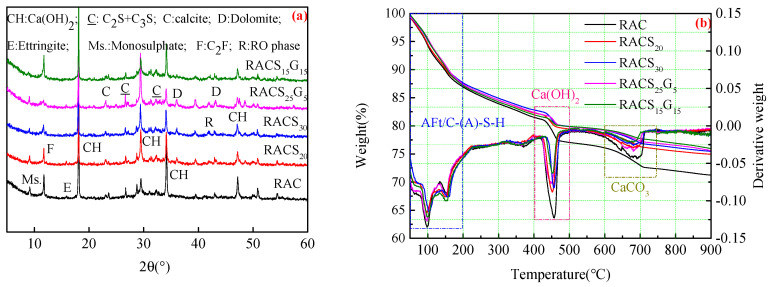
Curves for (**a**) XRD and (**b**) TG and derivative thermogravimetry (DTG).

**Figure 10:**
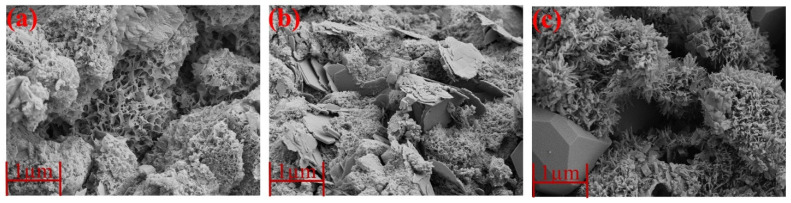
Morphologies of (**a**) RCA, (**b**) RCAS_30_ and (**c**) RCAS_15_G_15_.

To investigate the action of the inclusion of SS/GGBS on the hydration products of cement, five representative blended cement pastes at 90 days were used to analyze the types of hydration products. The XRD patterns of the SS/GGBS-influenced cement pastes are displayed in Figure 9a. The main hydration products included CH, ettringite (E), monosulfate (Ms), and calcite; unhydrated components (C_2_S and C_3_S) were also detected. When SS was added alone, no clear difference was observed in intensities, indicating that there was little difference in crystalline hydration between SS and plain cement. Notably, the diffraction peaks of CH decreased with increasing SS content, whereas those of C_3_S and C_2_S increased. This is due to the well-crystallized states of C_3_S and C_2_S during the raw production phenomenon under natural cooling. When 5–20% GGBS was added, the diffraction peaks of CH, C_3_S, and C_2_S gradually decreased, indicating the depolymerization of tetrahedral [SiO_4_] and [AlO_4_] in SS and generation of the C-S-H gel [12]. Previous studies [17,46] demonstrated that GGBS possesses high activity owing to its more amorphous phase, which can promote the decomposition of the aqueous phases and the precipitation of C-S-H, forming a mesh structure. This mesh structure can provide an environment for the growth and diffusion of crystals, expediting decomposition of the aqueous phases and leading to a higher generation rate and more pronounced expression of the C-S-H gel. Figure 10 shows the morphologies of the hydration products in the cement paste with SS/GGBS. In addition, the peaks of the RO phase were detected, which increased with more SS content. It has been elucidated that including GGBS has little impact on RO phase hydration, consistent with previous conclusions that the RO phase exhibits chemical inertness property [14,47].

**Figure 11:**
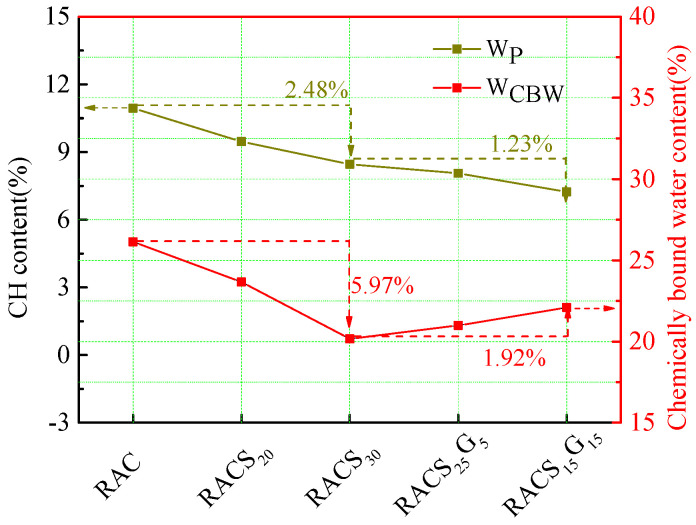
The content of CH and WCBW in pastes at 90 days.

The TG- DTG (derivative thermogravimetric) results of the SS/GGBS-influenced paste samples after 90 days are presented in Figure 9b. There are three obvious peaks of heat absorption at 50~200 °C, 400~500 °C, and 600~750 °C [48]. The endothermic peak at 50~200 °C is mainly attributed to the evaporation of free H_2_O and dehydration of Aft or C-S-H gel, and the peaks at 400~500 °C and 600~750 °C are primarily associated with the thermal decomposition of Ca (OH)_2_ (CH) and CaCO_3_, respectively. In Figure 9b, the portlandite (W_P_) and chemically combined water (W_CCW_) contents were measured using Equations (10)–(12), and are illustrated in Figure 11. The CH content decreased with an increase in the SS/GGBS content, which is in agreement with the result measured using XRD analysis. The content of W_CCW_ decreased as the substitution of SS increased, whereas it increased with the replacement of GGBS. Combined with the XRD results, it was further confirmed that GGBS contributes to the dissolution of anhydrous phases and the formation of the C-S-H gel, which improves the hydration reaction of SS and the mechanical properties of RACSG.

(10) $$W_{P}=\frac{74}{18}\times \left(\frac{W_{380{}^{\circ}C}-W_{450{}^{\circ}C}}{W_{50{}^{\circ}C}}\right)\times 100\%$$

(11) $$W_{\mathrm{CCW}}=\frac{\frac{W_{50{}^{\circ}C}-W_{900{}^{\circ}C}}{W_{50{}^{\circ}C}}-{\mathrm{LOI}}_{\mathrm{BC}}}{1-{\mathrm{LOI}}_{\mathrm{BC}}}\times 100\%$$

(12) $${LOI}_{BC}=\left(1-\alpha \right){LOI}_{C}+\alpha {LOI}_{S}+\beta {LOI}_{GGBFS}$$

where LOI represents the loss on ignition of raw materials, and α and *β* are the substitution of SS and GGBS for cement.

### 3.5. Effect of SS/GGBS and PP Fibers on Pore Structures of RAC

**Figure 12:**
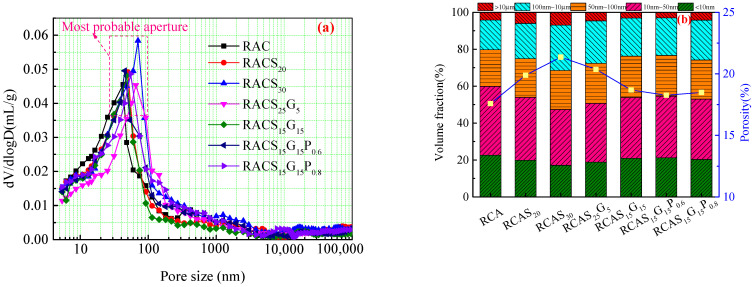
(**a**) The pore distribution and (**b**) the pore proportion distribution and porosity.

Figure 12 shows the pore structure results (cumulative porosity and pore distribution) of seven representative RAC samples in 90 days. The most probable aperture was a pore size manifested with an obvious pore peak, which conformed to the highest mercury infiltration point in the curves of the pore structures of RAC [49]. As shown in Figure 12a, the most probable aperture of the RAC was 42.64 nm, which increased to 70.57 nm with the addition of 30% SS, demonstrating that the incorporation of SS increased the pore size. In contrast, when both SS and GGBS were added, the most probable aperture of the RACS decreased, and a higher GGBS proportion resulted in a more significant refinement and a lower probable aperture. The most probable aperture for RACS_15_G_15_P_0.6_ was 45.1 nm, which was close to that of RACS_15_G_15_ (48.09 nm). Although the inclusion of an appropriate amount of PP fibers can optimize the pore structure by inhibiting the formation of microbubbles to a certain extent [50], adding PP fibers cannot change the amount and type of hydration products. Moreover, some hydration products are attached to the fiber surface, resulting in fewer hydration products in the matrix, excluding the PP fibers. Hence, the inclusion of PP fibers had little optimization effect on the pore structure of the RACSG. Meanwhile, it could be observed that the most probable aperture increased from 45.1 nm for RACS_15_G_15_P_0.6_ to 55.05 nm for RACS_15_G_15_P_0.8_, demonstrating that excessive fibers increase the bond interfaces in RACSGP, thus allowing connecting pores to be easily formed.

Pores in cementitious materials can be classified into five types according to size: gel pores ranging from 0 to 10 nm, harmless capillaries of 10–50 nm, harmful capillaries of 50–100 nm, harmful pores within 100 nm–10 μm, and severely harmful pores larger than 10 μm [20,51]. As displayed in Figure 12b, incorporating SS increased the porosity and proportion of harmful capillaries and pores in the RAC. Compared with RACS_30,_ RACS with GGBS showed a better optimization effect, which is attributed to the conversion of severely harmful pores into gel pores due to the pozzolanic effect of GGBS. Compared with RACS_30_, the percentages of pores below 50 nm in RACS_25_G_5_ and RACS_15_G_15_ increased by 7.11% and 13.82%, respectively. The pore refinement effect of RACS with GGBS was mainly observed for pores smaller than 50 nm. These results are similar to the conclusion drawn by Zhao et al. [17] that GGBS can significantly decrease porosity and lead to a higher proportion of pores below 50 nm. The porosity and percentage of pores below 50 nm in RACS_15_G_15_P_0.6_ were 18.26% and 54.59%, respectively, which were close to the values of 18.69%, and 53.84% in RACS_15_G_15_, respectively, indicating that the addition of PP fibers had little optimization effect on the pore characteristics of RACSG. However, RACS_15_G_15_P_0.6_ exhibited notably higher compressive and splitting tensile strengths than RACS_15_G_15_, which contradicts the conventional principle that a higher porosity and more harmful pores in concrete result in lower strength. This phenomenon is mainly attributed to the fact that the crack inhibition ability of PP fibers enhances its mechanical properties. The randomly distributed three-dimensional PP fiber network mitigates stress concentration at microcrack tips and restrains crack propagation to a certain degree, which effectively enhances the mechanical performance of RACSGP. Additionally, when the PP fiber content was 0.8%, the porosity and volume proportion of micropores below 50 nm in RACS_15_G_15_P_0.8_ decreased, revealing that an excessive amount of PP fibers was not conducive to optimizing the pore structure, as discussed in Section 3.3. Given the above functions of GGBS and PP fibers, the incorporation of suitable dosages of GGBS and PP fibers can improve the microstructural characteristics and macroscopic mechanical performance of RACS.

### 3.6. SEM-EDS Analysis

**Figure 13:**
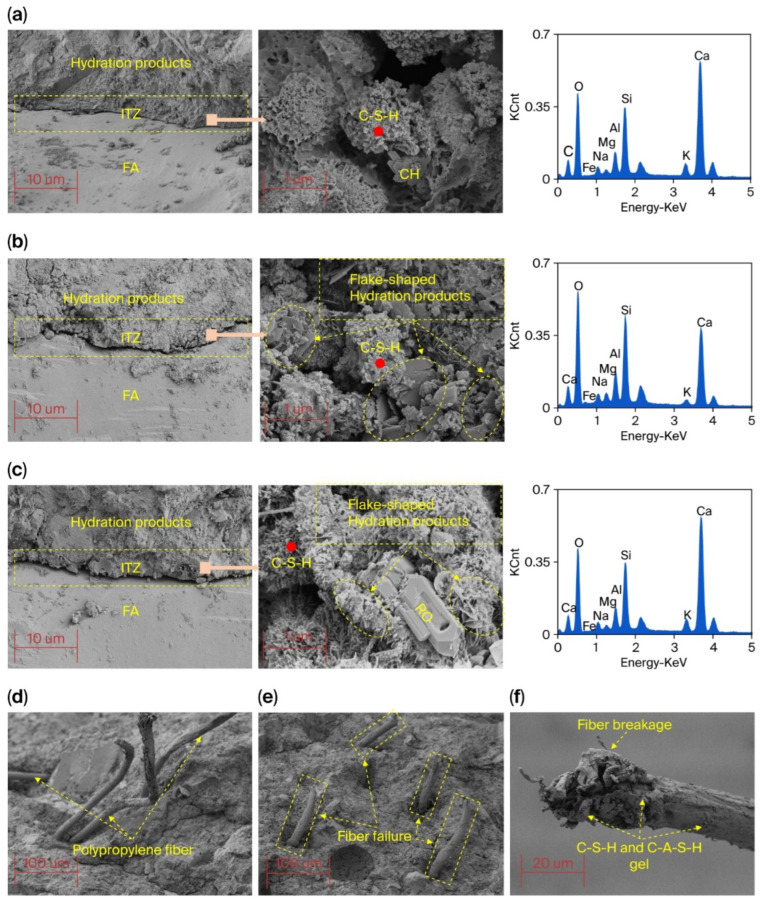
SEM images of (**a**) RAC (**b**) RACS_30_ (**c**) RACS_15_G_15_ (**d**–**f**) RACS_15_G_15_P_0.6_ at 90 days.

The ITZ is regarded as the weakest zone in ordinary concrete due to its high porosity. To demonstrate the impact of SS/GGBS and PP fibers on the RAC, SEM photomicrographs of RAC, RACS_30_, RACS_15_G_15_, and RACS_15_G_15_P_0.6_ are shown in Figure 13. Figure 13a shows that the ITZ of RAC is filled with homogeneous and dense hydration products, revealing that the hardened paste and RA bonded closely. When 30% SS was added, the microscopic ITZ (Figure 13b, RACS30) exhibited entrapped air voids, with visible cracks, and many flake-shaped hydration products. Therefore, the interfacial adhesion between the hardened paste and FA was poor, which significantly impacted the strength, porosity, and permeability of RACS. Meanwhile, RACS_30_ exhibited a Ca/Si ratio of 1.65 for the C-S-H gel, leading to a higher volume fraction of low-density C–S–H phases [52], which is unfavorable for the mechanical performance development of RACS. This coincides with the conclusions drawn from the mechanical properties and pore size distribution characteristics. The ITZ of RACS_15_G_15_ (Figure 13c) presented a relatively uniform and compact microstructure. The pozzolanic effect of GGBS promoted the hydration of cement and SS, generated more C–(A)-S-H gels, and refined the pore structure, thereby optimizing the interfacial zone. A large quantity of well-crystallized hydration products grew. Additionally, the incorporation of GGBS raised the Ca/Si ratio of RACS_30_ from 1.65 to 1.94 (RACS_15_G_15_), indicating the formation of high-density C-(A)-S-H gels and enhanced densification of the ITZ.

As illustrated in Figure 13d, PP fibers distribute randomly within the matrix and construct a three-dimensional network framework. They can withstand external loads from multiple orientations, thereby improving the mechanical performance of RACSGP. Figure 13e shows that one end of the PP fibers was embedded in the dense paste. The other end of the PP fibers was broken under the load, indicating that the PP fibers were tightly surrounded in the cement paste, and the bonding interface between the paste and PP fibers seemed to be strong. Additionally, it could be seen that many C-S-H and C-A-S-H gels were deposited on the fractured PP fiber surface. This demonstrated that PP fibers not only formed a large mechanical bite force with the cement matrix but also increased the interfacial sliding friction resistance upon fiber extraction. PP fibers dispersed in RACSGP exert a reinforcing effect analogous to steel rebars, and their secondary micro-reinforcement can effectively improve the mechanical performance of RACSGP [53]. The GGBS and PP fiber enhancement mechanisms in RACS are summarized and discussed in Section 3.7.2.

### 3.7. Mechanisms

#### 3.7.1. Modification Mechanism of GGBS

The ITZ exhibited a thickness of roughly 9–51 μm and had a higher pore content than the concrete matrix, which is closely associated with the macro- and micro-properties of concrete [50]. With respect to the mortar adhering to RA surfaces, the number of ITZs in the RAC was greater than that of regular concrete. Consequently, RAC exhibits inferior mechanical performance and durability compared with ordinary concrete. This is mainly because the adhered mortar of RA is characterized by high porosity, high water absorption, low density, and high surface and internal microcracks [54]. However, the incorporation of GGBS and SS densified the ITZ of the RAC to a certain extent by decreasing its porosity and changing its microscopic characteristics. As shown in Figure 1, SS and GGBS particles are significantly finer than cement particles, which not only fill the pores in ITZ-old (a loose, thin layer between the RA and adhered mortar, ITZ-O) but also those in ITZ-new (a thin layer between the RA and RAC matrix, ITZ-N) during the mixing and hardening processes of the RAC. The pore filling impact of SS and GGBS resulted in numerous nucleation sites in ITZ-O and ITZ-N, which were conducive to promoting cement hydration, improving the interfacial density, and decreasing Aft and CH enrichment on the RA surface by impacting the ion migration mechanism.

**Figure 14:**
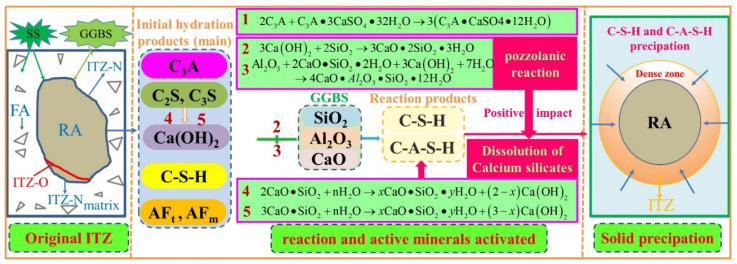
Reaction processes of GGBS enhancing ITZs [13].

Given the weak hydraulic activity of SS, the impact of the ion migration mechanism of ITZ-O and ITZ-N in the RAC is primarily attributed to the reaction of the GGBS, which involves five critical steps for enhancing ITZ-O and ITZ-N, as shown in Figure 14. First, sulfate ion consumption dramatically leads to the reaction of GGBS and C_3_A in the early stage (Figure 14, Equation (1)), producing a more stable monosulfate phase, which improves interfacial bonding and cohesion between RA and the matrix. Notably, more C-S-H and C-A-S-H gels are produced by the pozzolanic reaction between Ca (OH)_2_ and SiO_2_ and Al_2_O_3_ (see Equations (2) and (3) in Figure 14), which helps increase the Ca/Si ratio of ITZ-O and ITZ-N in accordance with the SEM-EDS analysis. Second, the pozzolanic consumption of Ca (OH)_2_ further promotes the dissolution of residual unhydrated C_2_S and C_3_S phases from SS and cement, as verified using XRD analysis. Third, the subsequent hydration degree of SS and cement is further promoted in the later period. Fourth, the continuous hydration of the cement–SS system effectively accelerates the generation of C-S-H gels (see Equations (4) and (5) in Figure 14). Finally, the C-S-H (C-A-S-H) gel particles are about 10–30 nm in size [55], which effectively transforms harmful capillary pores (50–100 nm) and harmful pores (100 nm–10 μm) into harmless capillary pores (10–50 nm) and gel pores (0–10 nm) via the continuous deposition of C-S-H (C-A-S-H) gels formed in continuous hydration (see Equations (2)–(5) in Figure 14).

The GGBS pozzolanic effect effectively reduced the diffusion of SO_4_^2−^, Ca^2+^, and Al^3+^ ions toward RA surfaces, leading to the transferring of SO_4_^2−^, Ca^2+^, and Al^3+^ from the aqueous solution to the surface of GGBS and the generation of abundant hydration products thereon, which enhanced ITZ-O and ITZ-N by alleviating CH enrichment on the interfacial surface. CH exerts a detrimental effect on the mechanical performance of concrete, as it is susceptible to fracture and tends to induce initial cracks around the ITZ under external loading [56]. Therefore, benefiting from the five aforementioned effects, GGBS addition efficiently optimized the pore characteristics of the RAC blended with SS. Additionally, adding GGBS scarcely affected the hydration behavior of the RO phase in the cement–SS composite system, as evident by the diffraction peak and morphology of the RO phase shown in Figure 9a and Figure 13c, respectively, indicating that the RO phase remains chemically inert despite GGBS incorporation. Moreover, due to the steaming process of SS [57], mechanochemical activation of SS [13], and previous results [14,33], the f-CaO in SS was removed substantially. As a result, the volumetric stability of the RAC blended with SS and GGBS gradually stabilized within seven days and complied with relevant specification demands. Therefore, GGBS incorporation benefits the mechanical performance and microstructural characteristics of RAC blended with SS.

#### 3.7.2. Function Mechanism of PP Fiber

**Figure 15:**
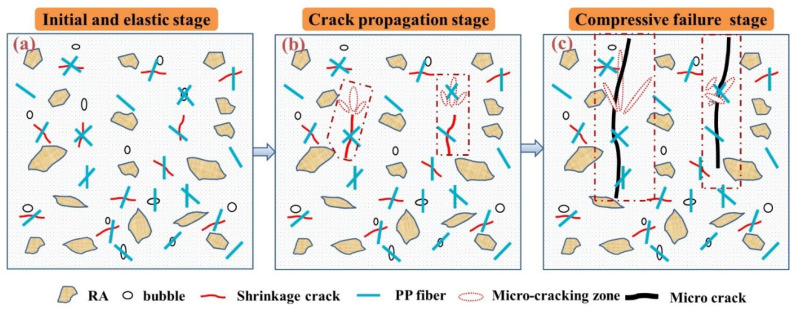
Schematic representation of RACSGP at different loading stages (**a**) initial and elastic stage (**b**) crack propagation stage (**c**) compressive failure stage.

Because the incorporation of PP fibers did not affect the hydration of the RAC containing SS and GGBS, the pore structure of RACSGP, especially the gel and capillary pores, according to the MIP (Figure 12b) results, would not be refined significantly. Since the added PP fibers did not fill the pores, the impact of PP fibers on RACSGP was mainly related to the macro-physical properties. As presented in Figure 15, the enhancement mechanism of PP fibers on RACSGP is divided into four major stages. First, PP fibers possess a high elastic modulus and are evenly dispersed in RACSGP, which is favorable for inhibiting the development of shrinkage cracks to a certain extent during the hardening process of RACSGP [58]. PP fibers play a restraining role in crack initiation and propagation through internal stress redistribution, alleviating the stress concentration at the microcracks and increasing the ultimate tensile strain, which contributes to the improvement of mechanical properties, namely the elastic stage. When the confining pressure increases, some cracks and bubbles that were inevitably generated during the hardening process expand and propagate, and new cracks form, as seen in Figure 15b. The high elastic modulus PP fibers are conducive to reinforcement bridging between the cracks and the concrete matrix by weakening the confining pressure concentration in the vicinity of the cracks and bubbles, which is the initial crack propagation stage. With a further increase in the confining pressure, the PP fibers cannot maintain a steady state and exhibit bending fracture (pulled-off or pulled-out), resulting in the transformation of microscopic cracks into macroscopic cracks (Figure 15c). The morphological characteristics of the broken PP fibers (Figure 13e,f) indicate that the interface adhesion between the matrix and PP fibers is strong, increasing the slip resistance under confining pressure. Correspondingly, the mechanical properties, particularly the splitting tensile strength, improve significantly. However, when an excessive amount of PP fibers was added, the PP fibers became prone to uneven dispersion and some large bubbles appeared in RACSGP in the mixing stage. Consequently, the compactness of RACSGP decreases, and the initial cracks and larger pores deteriorate. Therefore, from the above analysis, it can be concluded that when a 0.6% volume of PP fibers is added, RACSGP possesses high mechanical properties.

### 3.8. Development of GA-ANN Model

#### 3.8.1. Training the GA-ANN Model and Prediction of Compressive Strength

**Figure 16:**
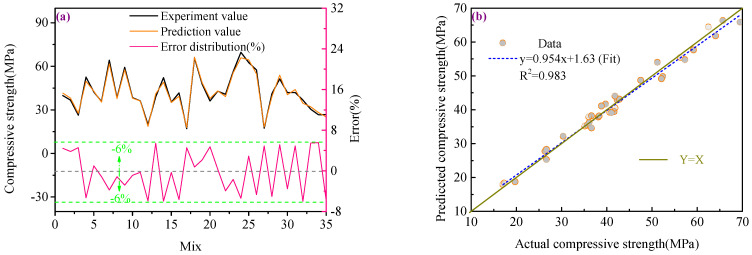
Evaluation of predicting compressive strength (**a**) the error between experiment value and prediction value (**b**) the regression curve.

A database of 135 experimental specimens collected through a broad survey of the relevant published literature [28,59,60] was used as the dataset source for modeling. According to the mixtures considered in this experimental study, the input variables reflecting the essential features were OPC (kg), SS (kg), GGBS (kg), FA (kg), NA (kg), RA (kg), water-to-cement ratio W/C, PP fiber (volume fraction), and sand ratio (*x*_8_). Moreover, it is widely known that compressive strength is a fundamental parameter in design specifications. Concerning the split tensile strength, which was not provided in some studies [28,60], and the relationship between compressive and split tensile strengths, which could be calculated based on formulas suggested by the Chinese code, the compressive strength was selected as the only output result. In this study, the training sets included 100 random datasets, and the remaining datasets were used to test the data. After repeated parameter adjustments, the GA-ANN model, which has a topological structure of 9-20-1 and 25 epochs completed in the network model training, was built to evaluate the compressive strength of RAC blended with SS, GGBS, and PP fibers. The numbers of evolutionary algebra, hidden layers, and epochs were 100, 20, and 25, respectively. This study adopted the fitness proportionate selection method. The selection probability of each individual for the next generation was directly proportional to its fitness value. In the Genetic Algorithm, genes at corresponding loci on two chromosomes undergo crossover to produce new chromosomes and further generate high-quality individuals. The crossover probability generally ranges from 0.10 to 0.99, and was set to 0.70 in this study. The probability of mutation was 0.10. The variation comparison between experimental and predicted data using the GA-ANN model is presented in Figure 16a, where the R^2^ of the training is 0.9337, confirming its precision in the prediction of compressive strength. Generally, a high R^2^ value is an essential parameter for estimating the accuracy of a good regression. To further assess the regression-based model, the error distribution between the experimental and test results was calculated using Equation (13) and plotted in Figure 16a, which shows that the maximum error is less than 6%. In addition, the experimental and training results are concentrated around Y = X (Figure 16b), indicating a good regression. Therefore, predicting the compressive strength of the RAC incorporating SS, GGBS, and PP fibers using the GA-ANN is feasible, practical, and achievable.

(13) $$Error\left(\%\right)=\frac{\left(Y_{i,exp}-Y_{i,pre}\right)}{Y_{i,exp}}\times 100\%$$

#### 3.8.2. Comparing the GA-ANN Model with Other Prediction Methods

**Figure 17:**
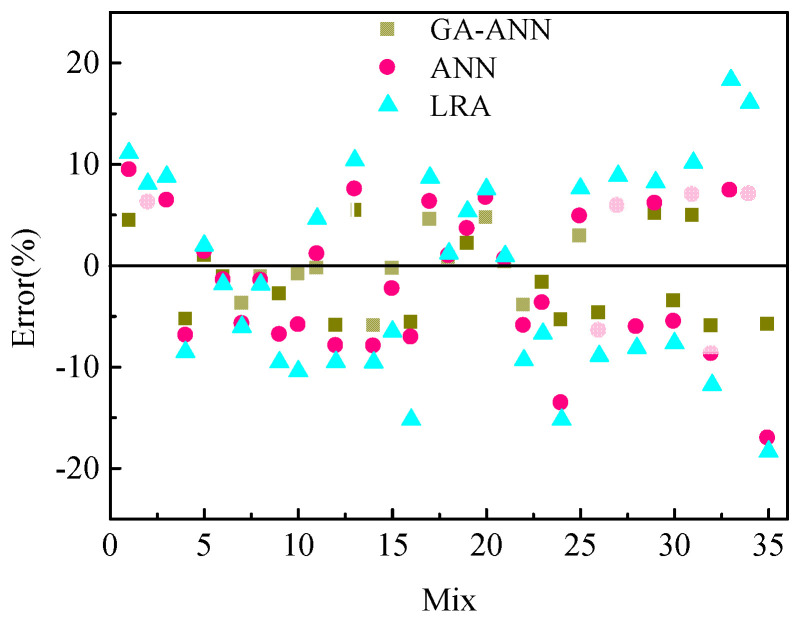
Comparison of error distribution obtained with different prediction models.

Recently, several predictive techniques, such as linear regression analysis (LRA), ANN models, and GA-ANN models, have been presented within the scope of computer-based artificial intelligence. To evaluate the predictive performance of these models, a comparative analysis was conducted between the GA-ANN, LRA, and ANN models. The prediction error distributions of the three models are presented in Figure 17. The GA-ANN model exhibited lower error distributions than the LRA and ANN models. It is worth noting that the LRA model is unsuitable for predicting the compressive strength of RAC incorporating SS, GGBS, and PP fibers because the more changeable and complex RAC mixtures create difficulties in linear and non-linear fitting. Moreover, we compared the precision of the GA-ANN model with that of the ANN using the correlation coefficient (R^2^), mean absolute percentage error (MAPE), and mean squared error (RMSE) calculated using Equations (14)–(16), and the results are summarized in Table 3 in detail.

(14) $$R^{2}=1-\frac{\sum_{i=1}^{N}{\left(Y_{i,pre}-Y_{i,exp}\right)}^{2}}{\sum_{i=1}^{N}{\left(Y_{i,exp}\right)}^{2}}$$

(15) $$MAPE=\frac{100}{N}\times \sum_{i=1}^{N}\left(\frac{Y_{i,pre}-Y_{i,exp}}{Y_{i,exp}}\right)$$

(16) $$RMSE=\sqrt{\frac{\sum_{i=1}^{N}{\left(Y_{i,pre}-Y_{i,exp}\right)}^{2}}{N}}$$

Table 3 shows that the accuracy and goodness of fit of the GA-ANN model are superior to those of the ANN model. For example, the R^2^ value of the ANN model was 0.8365, while the R^2^ of the GA-ANN model was 0.9337, an increase of 11.62%. This finding directly demonstrates that the GA-ANN model achieves a higher fitting accuracy than the conventional ANN model. Meanwhile, the RMSE and MAPE indicators of GA-ANN model were lower than those in the ANN model, which shows that the GA-ANN model possesses a higher fitting precision and smaller volatility of the fitting dataset. There are two reasons for this. On the one hand, GA and ANN algorithms can fully learn the advantages and characteristics of bio-neural networks and genetic evolutionary processes owing to their bionics. On the other hand, the GA can overcome the shortcomings of the ANN model by using search strategies and gradient-free global optimization inspired by genetic evolution processes, which is capable of optimizing the weights and thresholds of the ANN to minimize the deviation between predicted and actual values. From the above discussion, it can be seen that the precision of the GA-ANN is superior to that of the other models in terms of accuracy and flexibility, which offers the ability to estimate the compressive strength of RAC incorporating SS, GGBS, and PP fibers based on the available test results.

#### 3.8.3. Mix Design of RACSGP Based on GA-ANN Model

The utilization of GA-ANN in RACSGP mixture optimization design can not only decrease the consumption of human and material resources in the preliminary trial allocation but also add test results to retrain the network further, resulting in the continuous accumulation of datasets, thereby improving the accuracy and scope of application of network prediction. The relevant steps are as follows:

(1) Based on the current experimental database, the GA-ANN model was trained to predict the compressive strength of RACSGP.
(2) The RACSGP parameters in the input layer were determined by the appropriate boundary conditions of the raw material content, and the network predicted the corresponding performance parameters.
(3) The relationships between the raw materials and their corresponding performances were established by combining experimental data and predicted results.
(4) With the designed compressive strength as the goal, the preliminary mix design of the RACSGP could be determined using a reverse design based on the correlation established in step (3).
(5) The RACSGP was prepared according to the designed mixture, engineering requirements, and cost control.

**Figure 18:**
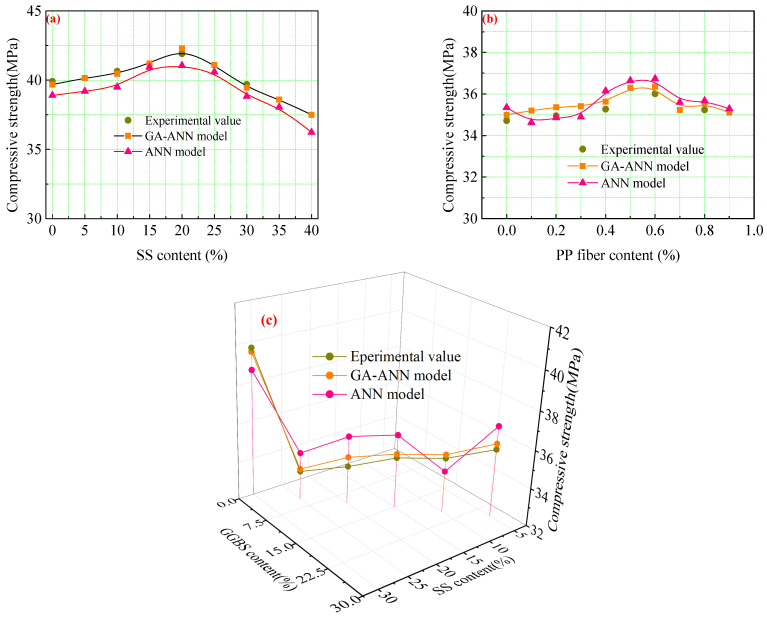
Examples of univariate and two-factor variable control methods (**a**) RAC with SS (**b**) RAC with SS and GGBS (**c**) RAC with SS GGBS and PP fibers.

To make the prediction model more applicable to the mix design of RACSGP, the measured values in the data samples and the predicted values of the neural network were taken as examples to analyze the regulation methods of different factors in the design. Figure 18a illustrates the impact of SS on the strength of RACSGP, with the other parameters unchanged. Notably, the GA-ANN model can accurately predict the influence of SS on the compressive strength of RACSGP, indicating that an appropriate SS content is beneficial to improving the compactness and strength of RAC. However, a higher content (more than 20%) adversely affects its strength. Therefore, the SS content in the RACSGP design can be inversely determined from the predicted curve and target strength. For example, when the target strength is 40 MPa, the SS content can be preliminarily determined to be approximately 10–27.5%, and then mix design and test verification can be performed. Moreover, Figure 18b illustrates the impact of PP fibers on the compressive strength of RACSGP, with other parameters unchanged. It can also be observed that the GA-ANN model can precisely estimate the effect of adding PP fibers on compressive strength, revealing that the optimized PP fiber content is 0.6%. Similarly, the PP fiber content in the RACSGP mixture can be reverse-designed based on the target strength and prediction curve. Figure 18b shows that including 0.5% PP fibers allows the development of a 35 MPa RACSGP in a preliminary mix design. Additionally, the impact of the other two factors, SS and GGBS, on the compressive strength of RACSGP were investigated and are presented in Figure 18c, revealing that the GA-ANN model can accurately estimate the strength influenced by multiple factors. According to the training graph (Figure 18c), suitable amounts of SS and GGBS can be determined to meet the target strength, and then the mix design and test verification can be conducted. Finally, although this study demonstrates the application of the GA-ANN model for predicting the compressive strength of RACSGP, the existing software cannot meet the demand for practical and convenient use of this technique. Therefore, the software should be perfected and supplemented with a more complete dataset and mix design in future studies.

## 4. Conclusions

In this research, the influence of SS, GGBS, and PP fibers on the mechanical properties and microstructural features of RAC were examined, and a GA-ANN model was established for the prediction of RAC compressive strength. Based on the experimental and simulation results, the main conclusions are summarized as follows:

(1) Owing to the low hydration activity of SS and GGBS at the early age, replacing OPC with SS and GGBS increased the workability of the RAC, while the addition of PP fibers had the opposite effect.
(2) Before 28 d, the substitution of OPC with SS and GGBS decreased the mechanical behavior of the RAC, except for the RAC samples containing 10% SS. After 28 days, the strength of all RAC samples increased with curing age, and substituting SS with GGBS significantly compensated for the negative impact of SS on the RAC strength.
(3) When the PP fiber content was less than 0.6%, RACSGP exhibited mechanical properties comparable to those of the reference group. The optimal PP fiber content of RACSGP was 0.6% with respect to the slump and mechanical properties of RACSG.
(4) The microscopic test results show that GGBS consumed some CH to form C-(A)-S-H gels, which benefits ITZ compactness and refines the RAC pore structure. The addition of an optimal proportion of PP fibers decreased the porosity of RACSG.
(5) A GA-ANN model was proposed for predicting the compressive strength of the RAC containing SS, GGBS, and PP fibers. The R^2^, RMSE, and MAPE were 0.9337, 3.4553%, and 5.776%, respectively, which proved the accuracy of the GA-ANN model in predicting RAC strength.

## Figures and Tables

**Table 1:** Chemical and physical properties of raw materials g (wt.%) [13].

Chemical Composition	SiO_2_	CaO	Fe_2_O_3_	Al_2_O_3_	K_2_O	SO_3_	MgO	P_2_O_5_	Others	Specific Surface Area (m^2^/kg)	Ignition Loss (%)
SS	18.80	65.83	3.35	5.15	1.22	2.95	0.92	0.44	4.41	560	0.0395
GGBS	14.81	44.78	25.48	4.08	0.05	0.48	4.34	1.57	1.2	416	0.0266
Cement	30.01	43.67	0.23	15.62	0.29	1.66	7.02	0.30	1.34	336	0.015

**Table 2:** Mix proportions of RAC (kg/m^3^).

Mixture	FA	CA	OPC	SS	GGBS	WG	W	PP (%)	PBS
RAC	683.4	1163.6	396	0	0	0	166.5	0	3.96
RACS_10_	683.4	1163.6	356.4	39.6	0	1.584	165.48	0	3.96
RACS_20_	683.4	1163.6	316.8	79.2	0	3.618	164.46	0	3.96
RACS_30_	683.4	1163.6	277.2	118.8	0	4.752	163.44	0	3.96
RACS_25_G_5_	683.4	1163.6	277.2	99	19.8	4.752	163.44	0	3.96
RACS_20_G_10_	683.4	1163.6	277.2	79.2	39.6	4.752	163.44	0	3.96
RACS_15_G_15_	683.4	1163.6	277.2	59.4	59.4	4.752	163.44	0	3.96
RACS_10_G_20_	683.4	1163.6	277.2	39.6	79.2	4.752	163.44	0	3.96
RACS_15_G_15_P_0.2_	683.4	1163.6	277.2	59.4	59.4	4.752	163.44	0.2	3.96
RACS_15_G_15_P_0.4_	683.4	1163.6	277.2	59.4	59.4	4.752	163.44	0.4	3.96
RACS_15_G_15_P_0.6_	683.4	1163.6	277.2	59.4	59.4	4.752	163.44	0.6	3.96
RACS_15_G_15_P_0.8_	683.4	1163.6	277.2	59.4	59.4	4.752	163.44	0.8	3.96

RAC containing SS (RACS); RAC containing SS and GGBS (RACSG); RAC containing SS, GGBS and PP fibers (RACSGP).

**Table 3:** Comparison of the performance of GA-ANN and ANN.

Evaluation Index	R^2^	RMSE	MAPE
GA-ANN	0.9337	3.4553	5.776%
ANN	0.8365	5.2145	8.657%

## Data Availability

The original contributions presented in this study are included in the article. Further inquiries can be directed to the corresponding author.
